# Nutrition promotion approaches preferred by Australian adolescents attending schools in disadvantaged neighbourhoods: a qualitative study

**DOI:** 10.1186/s12887-015-0379-7

**Published:** 2015-05-19

**Authors:** Lena D. Stephens, Sarah A. McNaughton, David Crawford, Kylie Ball

**Affiliations:** Centre for Physical Activity and Nutrition Research, Deakin University, 221 Burwood Highway, Burwood, VIC 3125 Australia

**Keywords:** Adolescent health, Social determinants of health, Nutrition

## Abstract

**Background:**

Links between socioeconomic disadvantage and unhealthy eating behaviours among adolescents are well established. Little is known about strategies that might support healthy eating among this target group. This study aimed to identify potential strategies and preferred dissemination methods that could be employed in nutrition promotion initiatives focussed on improving eating behaviours among socioeconomically disadvantaged adolescents.

**Methods:**

Semi-structured interviews were conducted in 2011 among 22 adolescents (12–15 years) recruited from secondary schools in disadvantaged neighbourhoods in Victoria, Australia.

**Results:**

Strategies suggested by adolescents to support healthy eating included increasing awareness about healthy eating; greater cooking involvement; greater parental and peer support; frequent family meal participation; greater parental and peer role-modelling of healthy eating; increased availability of healthy foods and decreased availability of unhealthy foods in homes and schools. Adolescents preferred electronic media, adolescent-specific recipe books, and school-based methods for distributing nutrition promotion messages and strategies.

**Conclusions:**

A number of suggested strategies and methods identified in the present investigation have been employed with success in previous nutrition promotion interventions targeting socioeconomically disadvantaged adolescents. The present study also contributes novel insights into potential strategies and methods that could be employed in initiatives aiming to improve eating behaviours in this vulnerable group, and particularly highlights the importance of incorporating strategies involving parents and modifying the home food environment.

## Background

Adolescents experiencing socioeconomic disadvantage (e.g. those from families with low levels of parental education, low income, or residing in socioeconomically disadvantaged neighbourhoods) are often shown to consume diets less consistent with dietary recommendations for good health when compared to more advantaged adolescents [1–3]. Socioeconomically disadvantaged adolescents are more prone to developing nutrition-related disease risk factors when compared with more advantaged adolescents [4].

To date, interventions aimed at improving diet conducted among adolescents from all SEP levels have not reported findings stratified by SEP, making it difficult to determine the effectiveness of such interventions among disadvantaged adolescents in comparison to those who are more advantaged. However, interventions may unintentionally result in widening socioeconomic disparities in diet [5]. For example, among adults, while a reduced-pricing intervention resulted in improvements in nutritional quality of foods purchased by disadvantaged women (who chose greater quantities of less healthy foods at baseline compared with more advantaged women), overall improvements were significantly lower among disadvantaged women than more advantaged women [5]. It is difficult to determine if dietary interventions focused on adolescents result in similar increases in socioeconomic disparities in diet as past research has not reported intervention findings stratified by SEP. However, the study conducted by Darmon and colleagues [5] suggests there is need for messages and strategies aimed at improving dietary intakes to be tailored specifically for socioeconomically disadvantaged groups.

A small number of interventions aimed at improving dietary intakes among adolescents of low SEP have been conducted, achieving varying degrees of success [6–11]. Those that have achieved favourable changes in disadvantaged adolescents’ eating behaviours have incorporated a multi-faceted approach to changing dietary behaviour through targeting intrapersonal (e.g. raising awareness of current eating behaviours [6–8], nutrition knowledge [7–9], goal setting and improving behavioural capabilities [10]), social (e.g. peer role-modelling of healthy eating [7, 8] and communicating about healthy eating with an individual who showed interest such as friends, parents [6, 8]), or environmental (e.g. the school food environment [11]) constructs and mediators.

Frenn and colleagues conducted three school-based interventions that drew on Health Promotion/Transtheoretical Model constructs and aimed to improve disadvantaged adolescent diet and physical activity [7–9]. In each intervention, strategies were tailored to adolescents’ stage of change (pre-contemplation, contemplation, preparation, action, and maintenance). The first intervention included four class-room sessions that focussed on raising awareness, nutrition knowledge and peer role-modelling, and resulted in a significant decrease in percentage fat in diet post-intervention [7]. The second intervention built on the first by including Internet and video formats, two additional school laboratory-based sessions, and content that aimed to increasing parental support for healthy eating [8]. That intervention resulted in a reduction in percentage dietary fat consumed by disadvantaged adolescents, with greater reductions amongst those completing more sessions [8]. A similar dose–response reduction in disadvantaged adolescents’ consumption of dietary fat resulted from the third intervention that extended on the previous two by incorporating two more classroom sessions [9].

In 2008, Di Noia, Contento and Prochaska described a successful CD-ROM intervention based on the Transtheoretical model that improved disadvantaged adolescents’ diet using tailored processes of change strategies, e.g. raising adolescent awareness about and peer support for healthy eating, based on adolescents’ stage of change [6]. Adolescents in the intervention arm had significantly higher daily fruit and vegetable servings post-intervention. Contento and colleagues’ 2010 curriculum-based intervention focused on increasing behavioural capabilities to improve diet among disadvantaged adolescents, and resulted in decreased serving size and frequency of consumption of high calorie beverages and packaged/processed snacks, and smaller serving sizes of fast food, but no changes in fruit, vegetable or water consumption [10]. Bere, Veierod and Klepp (2005) successfully improved disadvantaged adolescents’ fruit consumption by providing free fruit at school [11].

While the majority of these interventions successfully improved disadvantaged adolescents’ intake of a single dietary outcome, there remains a need to examine potential targets for improving disadvantaged adolescents’ diet across a range of food groups simultaneously in order to improve the impact of such interventions. The interventions described above also did not explore the effect of modifying adolescents’ home food environment, which can significantly improve adolescent eating behaviours [12]. Therefore further qualitative research is needed to identify relevant potential targets for nutrition interventions that draws on Social Ecological theories aimed at improving overall diet in this population.

Drawing on Social Ecological theories [13, 14], a number of observational studies have reported on correlates cross-sectionally and longitudinally associated with healthy eating among socioeconomically disadvantaged adolescents [15–21]. Intrapersonal factors associated with healthy eating among disadvantaged adolescents included greater perceived importance of health [16, 20], increased self-efficacy for increasing fruit consumption [15, 16] and reducing consumption of unhealthy food [16], healthy eating goal setting [17], fewer perceived barriers to consuming fruit and vegetables [17], more perceived benefits of consuming fruit and vegetables [17], increased involvement in cooking tasks [21], and infrequent consumption of high-energy foods for meals [19]. Social factors supportive of disadvantaged adolescents’ healthy eating including increased peer support for healthy eating (boys) [20], increased maternal role-modelling of healthy eating [16], and increased family support for healthy eating [16], with contrasting sex differences observed for adherence to family meal time rules (less stringent among boys, more stringent among girls) [20]. Increased access and availability of nutritious foods at home and decreased availability of high-energy foods at home were environmental factors strongly predictive of healthy eating among disadvantaged adolescents [16, 18–20].

How these potential intervention levers previously identified in observational studies [15–21] could be successfully adopted and implemented among disadvantaged adolescents and their families requires investigation. The format in which such nutrition promotion messages and strategies could be disseminated among disadvantaged adolescents and their families also needs exploration.

The present study aimed to identify potential strategies and preferred dissemination methods of nutrition promotion initiatives among socioeconomically disadvantaged adolescents. Gaining such insights will inform more relevant and practicable initiatives that could be implemented in interventions to increase their success in improving disadvantaged adolescents’ eating behaviours.

## Methods

### Participants

Adolescents were recruited from three co-educational Catholic secondary schools with enrolments ≥200. Catholic secondary schools were targeted for pragmatic reasons (i.e. due to time constraints on conducting the study it was deemed necessary to approach such schools as ethical clearance could be obtained promptly). Schools were selected from socioeconomically disadvantaged suburbs in metropolitan and non-metropolitan regions of Victoria, Australia. Suburbs were defined as disadvantaged if they ranked in the lowest two quintiles of the Australian Bureau of Statistics’ 2006 Socioeconomic Index for Areas (SEIFA) score of relative socioeconomic disadvantage [22]. Attributes such as the proportion of residents with low income, low levels of educational attainment and unskilled occupations are taken into account in the ranking [23].

In participating schools, adolescents enrolled in Year 7 (aged 12–13 years) and Year 8 (aged 14–15 years) were invited to participate. Younger rather than older adolescents were approached as past research has demonstrated that healthy eating behaviours established during early adolescence are likely to be sustained over time [18, 19]. Twenty-two adolescents (50 % girls) participated in telephone interviews.

### Procedure

To recruit adolescents, 8 eligible schools were initially posted an invitation to participate and were followed up by telephone to confirm interest in the study. Participating schools (*n* = 3) were sent a secondary school plain language statement and consent form, to be completed by the Principal. A total of *n* = 1501 (*n* = 744 Year 7 and *n* = 757 Year 8) students were shown a presentation about the study by the lead researcher during school assembly. Adolescents present on the day received a recruitment pack comprising a project introduction letter, adolescent and parent plain language statements and consent forms. Teachers were provided with additional recruitment packs to distribute among absentees.

Reminder notices were displayed around school one week after the school visit, and adolescents were given reminder letters two weeks post-visit. Participating schools received a $50 voucher for compensation for their time. Ethical approval was obtained from Deakin University’s Human Ethics Advisory Group (HEAG-H 22_2011) and the Catholic Education Office (GE11/0009 1690). Informed written consent was obtained from parents and adolescents, and appropriate steps were put in place to preserve confidentiality.

### Interview procedures

Upon receipt of completed parent and adolescent consent forms, parents were contacted by telephone to arrange bookings for interviews and to gather their sociodemographic data. All interviews took place over the telephone. Interviews were conducted individually as adolescents resided across a wide geographical area (metropolitan and non-metropolitan regions of Victoria, Australia), making it difficult to gather the participating adolescents for focus groups. Adolescent participants (*n* = 22, 1.5 % response rate) were informed that the interview would be audio-taped, information provided would remain confidential, and that they could withdraw from the study at any time. The interview schedule was pilot tested and refined with the first two adolescents, and as very limited suggestions regarding interview content were made (i.e. very minor modifications to wording, and no removal/addition of questions posed), pilot data were included in analyses described in the present investigation.

Participants were interviewed individually, and interviews lasted 33–60 min (average 44 min). To facilitate data analysis notes were taken during the interview. Adolescents continued to be recruited to the study (*n* = 22) until interview data showed saturation of content (i.e. adolescents were repeating themes that had been discussed previously in other interviews). Adolescents were provided a $10 voucher in recompense for their time. General results letters were posted to participating schools and adolescents three weeks after the conclusion of the study.

A study was conducted among parents of the adolescents participating in the present investigation, and strategies and preferred dissemination methods as identified by parents will be reported elsewhere.

### Semi-structured interview schedule

A semi-structured interview schedule (Table 1) was developed to include questions of original design covering broad topic areas about intrapersonal, social and environmental factors related to adolescent eating behaviours. Questions were based on Social Ecological theories [13, 14] and previous research examining correlates of and potential intervention strategies aimed at improving adolescent eating behaviours [18–20, 24–26].

**Table 1:** Adolescent interview questions investigating strategies supporting healthy eating and preferred nutrition promotion initiative dissemination methods

	Adolescent interview questions
Identification of strategies aimed at improving adolescent eating behaviours	Strategies to improve general eating behaviours:
	‘What do you think would help you/other adolescents eat breakfast every day?’,
	‘What would help you/other adolescents choose healthier options at a fast food restaurant?’, and
	‘What can you think of that could help you eat a healthy meal rather than fast food?’
	Strategies to improve eating behaviours at school:
	‘Can you think of things you/other adolescents could do to avoid buying snacks on the way to or from school?’, and
	‘Can you think of things you/other adolescents could do to avoid buying food/drink from the school canteen?’
	Strategies to improve perceived importance of healthy eating:
	‘How do you think other adolescents could be convinced that eating healthfully is important?’
	Strategies to increase self-efficacy:
	‘Can you think of some things that would make it easier for you/other adolescents to eat more healthfully at home/school/when hanging out with friends in places other than at school?’
	Strategies to increase cooking involvement:
	‘Can you think of some things that would help you/other adolescents cook more often?’
	Strategies targeting peers:
	‘What would your friends need to say to encourage you to eat healthy foods?’, and
	‘What could you/other adolescents do to encourage your/their friends to eat healthy foods?’
	Strategies to implement family meal time rules:
	‘If parents of other adolescents set meal time rules, what do you think parents would have to do to get their adolescent to follow those rules?’
Preferred avenues of disseminating nutrition promotion initiatives	Interest in receiving initiatives:
	‘Do you think you would be interested in receiving information about the things we talked about today? For example, about how to choose healthier meal options in place of fast food, or how to replace unhealthy foods at home? Why/why not?’, and
	‘Do you think other adolescents would be interested in receiving information about the things we talked about today? Why/why not?’
	Preferred avenues:
	‘How do you think you would like to receive this information, for example, leaflets, SMS, email, Facebook page or other social networking sites, etc.?’, and
	‘How do you think other adolescents would like to receive this information?’

Adolescents were prompted to discuss strategies for improving eating behaviours in a variety of settings. They were also asked to identify avenues for disseminating nutrition promotion messages and strategies targeting adolescents that they themselves preferred, as well as what they thought would be preferred by other adolescents.

### Sociodemographic characteristics

Parents reported their child’s sex, their own sex, relationship to the child, and highest level of schooling. They were also asked their relationship status and, if applicable, their partner’s relationship to the child and partner’s highest level of schooling. Sociodemographic characteristics were gathered for 22 adolescents, excluding partner’s relationship to the child and partner’s highest level of schooling for one participant whose parent did not have a partner. Equal proportions of girls and boys were recruited, the majority of adolescents were from metropolitan Victoria, and more adolescents were enrolled in Year 8 than Year 7. In 2011, the Year 7 and 8 Catholic school population in Australia was 50 % boys, and 40 % enrolled in Year 7 [27]. When compared with the wider Australian Catholic school population, similar proportions of boys and girls were recruited in the present investigation, with slightly more Year 7 students represented in the present sample. In terms of SEP (based on maternal education), only a small proportion of low SEP adolescents were recruited (<15 %), and 45 % of adolescents were of high SEP. Parental education data of the student body as a whole were not available therefore it is difficult to ascertain how representative the sample was in this regard.

Individual-level measures of SEP were based on parental education, a well-established proxy for adolescent SEP [24, 28–30]. This was derived from the highest level of maternal (the biological mother or female guardian) or paternal (the biological father or male guardian) education. The highest level of education attained was categorised into three groups: ‘low’, completed up to Year 10 of high school; ‘medium’, completed Year 12 high school and/or a technical or trade school certificate/apprenticeship; and ‘high’, completed a university or tertiary qualification.

### Data analysis

Interviews were digitally recorded and transcribed verbatim. At the end of each interview emerging themes were annotated to direct subsequent interviews, consistent with the principle outlined in grounded theory of qualitative research [31]. Transcripts were assigned a unique identification number to ensure confidentiality (e.g. A1=adolescent 1).

A manual qualitative data analysis method was employed by the lead researcher (L.S.) based on the raw transcripts, using a method entailing four key steps including immersion in the data, coding, creating categories, and the identification of themes [32]. Reading and re-reading of the interview transcripts and listening to interview recordings to build familiarity with the data enabled immersion in the data.

Inductive thematic analysis [33] was then used to code data using descriptive labels. Categories were formed by linking coded data together [32]. Major themes and concepts were linked to direct quotes. Illustrative quotes from adolescents selected to demonstrate responses which were common, contrasting or representing a summary of a topic, are provided in below with assignment of IDs and adolescent sex and year level.

## Results

Sociodemographic characteristics of the sample are summarised in Table 2. Half of participants were girls, 45 % were enrolled in Year 7, and 64 % were recruited from metropolitan secondary schools. The majority of adolescents (55 % based on maternal, 67 % based on paternal) had parents with low- or medium-level education.

**Table 2:** Sociodemographic characteristics of adolescents

Sociodemographic characteristics	Percent
Sex of adolescent	
Boys	50
Girls	50
Maternal education^a^	
Low	14
Medium	41
High	45
Paternal education^a,b^	
Low	14
Medium	53
High	33
Region of residence	
Metropolitan	64
Non-metropolitan	36
Year level of adolescent	
Year 7	45
Year 8	55

^a^Education: Low ≤ Year 10 high school, Medium = Year 12 high school/Trade certificate and High = tertiary education

^b^ *n* = 21 (one participating parent/carer did not have a partner)

Adolescents suggested a number of strategies as potentially useful for improving adolescent eating behaviours, with the most commonly cited strategies including increased availability and accessibility of healthy foods and decreased availability and accessibility of unhealthy foods and increased cooking involvement. Adolescents also often suggested needing parental and peer support to eat healthfully and needing to increase awareness about healthy eating. Other strategies that were cited less often, but identified as important, included regular participation in family meals and increased role-modelling of healthy eating. These main themes are described below grouped based on levels of the Social Ecological models.

### Strategies to improve adolescent eating behaviours

#### Increased awareness about healthy eating

Most adolescents reported strategies for increasing adolescent awareness about the importance of healthy eating. A major strategy suggested by participating adolescents for increasing adolescent awareness about the importance of healthy eating was to provide adolescents with education from parents and schools about the short- and long-term benefits of eating a healthful diet and consequences of consuming unhealthy foods, particularly employing ‘shock tactics’ to emphasise this.

“You have to scare [adolescents], and say, ‘If you don’t eat healthy you’re going to get some serious issues’, or something like that…. Because if you just tell them, they won’t do anything about it.” (Girl, Year 8–A4)

Adolescents also thought being given access to nutrition information could help them make informed choices about meals and snacks.

“Like if you’re aware of how many calories that are in [the fast food menu option], I’d usually choose the healthier option. Like compared to the unhealthy stuff.” (Girl, Year 8–A1)

#### Increased cooking involvement

A frequently occurring theme was the importance of cooking involvement as a strategy for improving eating behaviours among adolescents. The majority of adolescents identified several methods that could be used to involve them in cooking. The most commonly mentioned strategy was to make cooking enjoyable by adolescents choosing recipes such as healthy home-cooked versions of fast food items such as hamburgers and pizzas, or utilising favourite cooking methods like baking. Being able to cater to taste preferences and participating in all stages of meal preparation (e.g. menu planning, shopping, preparation and cooking) were also described as important. Cooking could also be made more fun if it was made an event for the whole family, for example as an alternative to purchasing fast food.

“Like we go to [name of supermarket] sometimes and pick out what I want for tea so I can help Mum make it or help out making it. And that’s fun for me.” (Boy, Year 7–A5)

Adolescents identified parents as key in getting adolescents involved in cooking. Some suggested that adolescent participation in cooking had to be made compulsory, and could be viewed as sharing the workload of preparing the family meal with parents. Parents could also be encouraged to reward adolescents for cooking; for example, allowing adolescents to do another enjoyable activity afterwards, or to trade, like not having to wash dishes if adolescents helped cook. It was also suggested that parents should actively teach their adolescent how to cook, encouraging and praising them in order to help them to enjoy cooking.

“If the parents made [adolescents] cook like one night a week. They could like give them a little reward or something, like for that night they could have an hour more on the computer or something. They could go to the movies, or if they cook for a whole month they could go to the movies.” (Girl, Year 8–A2)

Some adolescents also suggested that the school curriculum could be modified to ensure all adolescents in secondary schools are provided with opportunities to participate in school cooking lessons, as adolescents also thought participation in cooking at school would encourage them to cook more often.

“Maybe [adolescents] could try food [technology] at school.” (Boy, Year 8–A13)

#### Increased parental support to eat healthfully

The importance of parental support for healthy eating was commonly mentioned by all adolescents. It was suggested that greater communication and negotiation between adolescents and parents regarding eating healthfully should be fostered; for example meal choices could be discussed and negotiated in order to cater to adolescent taste preferences. Parents could offer adolescents verbal encouragement to choose healthy foods.

“Maybe like, because my Mum cooks really good things which are healthy at home, that are pretty delicious, so ask the kids like, ‘What do you want?’ instead of just making them steamed vegetables and all that, so you can have something else which is still healthy.” (Boy, Year 8–A3)

In order to help enforce family meal time rules, parents could be encouraged to follow the rules themselves, and reward adolescents’ adherence to the rules while punishing noncompliance.

“Be like, ‘You have to sit at the table or you can’t go on your laptop for tonight’, or something like that, and then you have no choice. You can’t live without laptops, us teenagers. Sort of bribe us. Maybe, like, I don’t know, getting a reward after it. Maybe [adolescents] know there’ll always be dessert after or something like that.” (Girl, Year 8–A4)

#### Regular participation in family meals

Some adolescents suggested frequent participation in family meals could also be promoted to families to support adolescents to eat healthfully.

“You have to eat at the dinner table, you can’t eat anywhere else because when you eat anywhere else except the dinner table you get distracted…” (Girl, Year 8–A11)

#### Increased peer support to eat healthfully

Adolescents expressed the view that support from the friends to eat healthfully would benefit their eating behaviours. Most adolescents suggested a number of strategies for increasing peer support to eat a healthful diet, including adolescents increasing one another’s awareness about the importance of eating healthfully, encouraging each other to eat a healthful diet, and providing nutritious foods to share with one another.

“Go through every step [of eating healthfully] with [my friends]… If they’re going to eat some junk food, just tell them to eat just a little bit, and then go back to like eating a banana.” (Boy, Year 8–A13)

#### Increased role-modelling of healthy eating

Several adolescents suggested the importance of role-modelling of healthy eating by adolescents’ parents, peers, and other people from whom adolescents could learn to eat healthfully by observation. Role-models could demonstrate the benefits of consuming a healthy diet, or the consequences of consuming a poor diet to convince them about the importance of eating a healthful diet. Parents and peers could also act as role-models of healthy eating for adolescents.

“Your family has a big influence on what you eat, so they should be setting a good example for [adolescents]” (Girl, Year 8–A10)

#### Increased availability and accessibility of healthy foods and decreased availability and accessibility of unhealthy foods

The most frequently suggested strategies to support adolescents to eat healthfully included those focused on increasing availability and accessibility of healthy foods while decreasing that of unhealthy foods. These included adolescents swapping unhealthy foods for healthy foods, parental facilitation, adolescents carrying healthy food and drink when away from home, parents ensuring healthy meals and snacks (or ingredients for these) are available at home when families were short on time, and parents and adolescents purchasing healthy alternatives instead of having fast food.

Nearly all adolescents thought swapping unhealthy foods for healthy foods in different settings, for example at home and in their school lunch, would be a simple method by which they could increase availability of and access to healthy foods.

“Take a piece of fruit or vegetables, instead of taking a packet of chips to school.” (Girl, Year 7–A16)

Adolescents also viewed it as their parents’ responsibility to facilitate availability and access to healthy foods by shopping for and preparing healthy foods for the adolescents to eat. Parents could also regulate adolescents’ access to unhealthy foods away from home by limiting the amount of spending money adolescents had.

“If [fruit and vegetables are] cut up or something and there, it’s ready to eat, I’ll eat it. If there was like less junk food and like only a few stuff of like chip bags and stuff.” (Girl, Year 7–A21)

Suggested school environmental changes included eliminating unhealthy foods from school canteens coupled with the provision of nutritious meals and snacks at a low cost to students.

“Well firstly [schools] should have more healthier options at the canteen, and they could have them on special or something… they could have days where they’re cheaper or something so it encourages people to buy them, and then they’ll taste it and see they like it and they’ll keep buying it.” (Girl, Year 8–A10)

Implementing nutrition promotion programs with which adolescents were familiar, such as the ‘Free Fruit Friday’ component of the ‘Kids–Go For Your Life’ initiative [34] could increase adolescents’ access to healthy foods at school.

“Well, at my primary school they used to have like a ‘Free Fruit Friday’ sort of thing and they’d bring out a huge bowl of fruit and everyone would dig in to that ‘cause it was there and then [adolescents would eat more fruit at school].” (Girl, Year 8–A8)

To combat lack of availability of healthy foods in places outside the home at times that adolescents may be hungry (e.g., at a friend’s house or the cinema), adolescents noted that they could be encouraged to carry healthy foods with them, or eat something healthy before they left home.

“Maybe if [adolescents] brought something like their food with them and they knew what they were going to eat, not just on the day decide what they’re going to eat out at the shops because they’re more tempted with food and junk food.” (Boy, Year 7–A22)

It was suggested that parents could ensure healthy foods were always available at home to cater for when the family did not have time to cook healthy meals or snacks. For example, parents could have ingredients for healthy meals in the refrigerator or pantry, or healthy meals prepared previously and stored in the freezer. Adolescents also suggested they and their parents should have access to recipes for quick and easy healthy meals.

“If food was more easier to prepare. I already know that [people] have the jars with the tomato sauce and all they have to do is put it in the pan with the meat and the pasta and that's pretty easy. So maybe if that was advertised more, like quick meals and stuff.” (Girl, Year 8–A14)

Rather than purchasing fast food, families could also be encouraged to prepare healthy versions of fast foods, e.g. hamburgers or pizzas; alternatively they could be encouraged to purchase a healthier meal option, e.g. charcoal chicken with a home-made salad.

“If you’re going to go have junk food like [name of fast food restaurant] or something, probably think, I’ll stay home, then you can make your own burger, a healthy burger.” (Boy, Year 7–A17)

### Preferred avenues for disseminating nutrition promotion messages and strategies aimed at improving adolescent eating behaviours

Participants described several ideas regarding the content, format of distributing nutrition promotion messages and strategies among adolescents. In terms of the content of nutrition promotion messages and approaches, adolescents felt it was important for initiatives to provide education about what constitutes healthy eating, dietary recommendations, reasons for eating healthfully, healthy eating tips, goal- and challenge-setting, recipe ideas for nutritious snacks and meals, and success stories. On Internet-based formats in particular adolescents wanted to access a forum where they could ask for nutrition advice, play educational games, and see online cooking demonstrations. Adolescents also wanted school canteens to promote nutritious foods around the school, e.g. by putting up posters advertising discounted healthy foods.

Adolescents wanted to voice their opinion and shape content in promotion materials, particularly those disseminated via electronic media. Also, they felt that information should be cost-free. Nutrition promotion materials needed to be bright, colourful, fun, interactive and concise.

Adolescents also identified a range of formats through which they could access nutrition promotion messages and strategies. Adolescents heavily favoured electronic media as preferred modes for accessing information, including websites (particularly Facebook, mentioned most frequently of all avenues discussed), YouTube videos, and advertisements on the internet, and email. Also mentioned were mobile phone messages via the Short Message Service (SMS), radio and television advertisements (including news bulletins), and magazine and newspaper advertisements in print media.

Adolescents commonly mentioned leaflets mailed home and recipe books specifically designed for adolescents. School was a popular site through which adolescents wanted to access nutrition information via curriculum (e.g. home economics classes which could be offered to adolescents at a younger age), posters and leaflets (made by the school student committee), newsletter notices, presentations by professionals (e.g. dietitian), presentations or plays conducted by adolescents’ peers of varying ages who eat healthfully (i.e. as role models), and also a healthy eating activity day.

## Discussion

In this study, adolescents from schools in socioeconomically disadvantaged neighbourhoods discussed a range of intrapersonal, social and environmental strategies which they perceived would be helpful in promoting healthy eating among adolescents. A variety of preferred content and modes of disseminating nutrition promotion messages and strategies aimed at supporting adolescents to eat well were also identified. Although adolescents identified strategies targeting intrapersonal, social and environmental influences on eating behaviours, environmental strategies were most often described by all participants, followed closely by strategies targeting social influences.

When SEP was defined using an individual-level measure of SEP (i.e. parental level of education), more participants were from advantaged backgrounds. Therefore the findings in the present investigation could be transferrable for implementation in nutrition promotion messages and strategies targeting adolescents from the wider population irrespective of SEP. These strategies have had previous success in improving adolescent diet across socioeconomic strata. For example, increased awareness about healthy eating [35], increased parental support to eat healthfully [36], increased peer support to eat healthfully [37], and increased availability and accessibility of healthy foods and decreased availability and accessibility of unhealthy foods at home [12] and at school [36].

Participants most frequently suggested increasing availability and access of nutritious foods while decreasing that of unhealthy foods in different settings to support healthy eating. Increases in access and availability of healthy foods at school was achieved by making fruit available in a peer-led group fruit snack session that resulted in improved fruit and vegetable intakes among disadvantaged adolescents [7]. No previous interventions targeting disadvantaged adolescents incorporated strategies aimed at changing the home food environment, however a previous pilot study among adolescents from all SEP levels showed that increased availability and accessibility of fruit and vegetables at home resulted in improved consumption of those foods [12], confirming these as important potential intervention targets for adolescents from all socioeconomic levels.

Adolescents described increasing availability and access of nutritious foods at school as a strategy to improve their eating behaviours. Changes to the school food environment can result in significant improvements to eating behaviours among disadvantaged adolescents [7, 8, 11] and adolescents from all SEP levels [38–41]. Dietary intakes of adolescents from all SEP levels have been shown to be improved by school food policy change [42–44]. Adolescents in the present investigation also felt nutrition promotion initiatives implemented during primary school could continue on into secondary school in order to support adolescents to eat a healthful diet. Interventions could target school environment and policy changes to support adolescents’ adoption of a healthy diet. Increasing availability and accessibility of healthy foods and decreased availability and accessibility of unhealthy foods at school shows promise in improving dietary intakes among adolescents irrespective of SEP [36]. Future research examining the feasibility of executing changes of this nature to policy and environment in this setting could involve investigation with key stakeholders (e.g. secondary school Principals, individuals within government departments and agencies).

Developing adolescent skills in food preparation could also be considered in intervention design. Making cooking an enjoyable activity was most commonly mentioned as a strategy to support disadvantaged adolescents to participate more often. Few previously successful interventions have employed participating in cooking to promote low-fat diets among disadvantaged adolescents [7, 8]. For example, only two interventions involved a single session each in which adolescents prepared healthy snacks in small peer-led groups in order to build self-efficacy, overcome barriers to eating healthy foods by fostering taste preferences and developing behavioural capacity to prepare such foods [7, 8]. Home economics could be reinstated in secondary schools as a compulsory component of the curriculum, since in the past it served to foster food preparation skills among adolescents [45]. Discussion of ingredients and sharing of recipes were included in three successful school-based interventions aimed at reducing dietary fat intakes of disadvantaged US adolescents [6–9], and past research has also demonstrated that disadvantaged adolescents are receptive to intervention strategies involving cooking classes [46–48].

Parents were also identified as key in getting adolescents involved in cooking. No previous interventions aimed at improving dietary intakes among adolescents have targeted parents along with adolescents in developing cooking skills. However, involving parents in such a strategy is reasonable, as adolescents have previously acknowledged their parents’ role in teaching them cooking skills. For example NZ adolescents from all SEP levels reported learning cooking skills from their mothers, either directly or by watching [49]. Interventions focused on development of cooking skills may need to incorporate cooking lessons for parents of disadvantaged adolescents, as there is some evidence that adults with lower education levels lack confidence to cook [50]. Cooking with friends was also identified as a potential strategy to promote disadvantaged adolescents’ participation in cooking, a strategy successfully employed by Frenn and colleagues [7, 8]. Parents, particularly those who only have limited time available, could increase their involvement in promoting healthy eating to their adolescents by including adolescents in all stages of meal preparation (e.g. meal planning, preparing a shopping list, doing the shopping, preparation and cooking). Such strategies could be integrated with other time-saving tactics for preparing meals (e.g. cooking dinner ahead of time the night before, or using a slow cooker), cooking foods in bulk and freezing excess for later meals, and purchasing pre-prepared healthy foods in packs (e.g. salad).

Results also suggest that greater communication and discussion between adolescents and parents regarding eating a healthful diet might be effective in improving eating behaviours among adolescents. Participation in family meals and adherence to meal time rules are also important aspects of such parent-adolescent communication. Interventions including communication about healthy eating with an individual who showed interest (e.g. parents) have been shown to be successful in increasing fruit and vegetables intakes [6] and reducing fat intakes [8] among disadvantaged US adolescents. Further, among disadvantaged adolescents, frequent participation in family meals is positively associated with frequent consumption of breakfast and daily fruit intake [51]. Youth proxy efficacy has been defined by Geller and Dzewaltowski [52] as an adolescent’s confidence to negotiate with and influence parents to purchase fruit and vegetables. Studies have shown that adolescent boys and those experiencing socioeconomic disadvantage had significantly lower levels of proxy efficacy when compared with adolescent girls and more advantaged adolescents, respectively [52]. Fostering participation in family meals has not previously been implemented in interventions targeting adolescents. Regular participation in family meals confers many benefits to adolescents, particularly in promoting healthy eating [53–55] and may provide an opportune avenue for fostering greater communication between adolescents and their parents [56, 57]. Therefore interventions could focus on promoting regular family meals to increase adolescent-parent communication about issues related to the home food environment.

Adolescents identified a number of preferred ways in which nutrition promotion messages and strategies could be disseminated among disadvantaged adolescents and their families. As expected, adolescents heavily emphasised their preferences for online and social media for delivery of such messages and strategies. Most frequently, adolescents discussed being able to access websites (particularly Facebook) that included a forum where they could ask for nutrition advice, play educational games, and see online cooking demonstrations (e.g. YouTube videos). A previous CD-ROM-based tailored intervention successfully engaged disadvantaged adolescents to improve their fruit and vegetable consumption [6]. Similarly, school-based interventions including Internet and video components resulted in decreased fat consumption among disadvantaged adolescents [8, 9]. The interventions conducted by Frenn and colleagues [8, 9] also incorporated some of the strategies identified by adolescents in the present investigation, including food preparation in peer-led sessions. Taken together, methods of dissemination as identified in the present investigation could be promising if incorporated into future nutrition promotion initiatives targeting adolescents from socioeconomically disadvantaged neighbourhoods.

### Strengths and limitations

A number of study limitations should be acknowledged. Possible participation bias may exist, in that participating adolescents may have been more interested in nutrition and health than non-participants. However, broad representativeness of the sample is not an aim of qualitative studies which rather intend to generate a range of responses and hypotheses that may be followed up in future studies. It also may be possible that during interviews adolescents provided socially desirable responses (e.g. describing having more favourable eating behaviours than they did in reality). However participants described many challenges faced in consuming healthy foods and openly discussed barriers to doing so, suggesting that socially desirable responses were minimised. Also, the use of one-on-one telephone interviews to collect data may reduce some forms of response bias as participants are less affected by cues from facial expressions or perceived social desirability from the researcher (e.g. in one-on-one interviews) or other participants (e.g. in a focus group setting) [58, 59]. Participants may also be more forthcoming with responses given the anonymity associated with telephone contact [58].

Although all participants were recruited from socioeconomically disadvantaged neighbourhoods, a small proportion were of high SEP based on maternal education (an individual-level indicator of SEP), which may impact the transferability of strategies suggested by participants to support adolescents’ healthy eating when applied to disadvantaged families in the wider population. Further, as parents were not asked to report their income, a more comprehensive description of adolescent SEP could not be provided. Repeated efforts were employed to recruit socioeconomically disadvantaged families by sampling from neighbourhoods defined as disadvantaged using an area-level measure (SEIFA), and while a proportion of participants had higher SEP when based on an individual-level measure, these participants would still face nutritional challenges associated with attending schools in disadvantaged neighbourhoods [60–62]. Catholic schools may differ from other schools, particularly in terms of the religious affiliations of students and their families. These schools also require a small tuition fee be paid by enrolled students, and adolescents from these schools may therefore be more advantaged than those from tuition-free government schools. Restricting recruitment to these schools only may represent a source of bias in the present investigation. However it was necessary to be pragmatic in recruiting as these were schools in which gaining ethical clearance was faster when compared to government-run schools. Despite these limitations, participants came from a range of backgrounds providing valuable insights into influences on disadvantaged adolescent eating behaviours and potential strategies to support healthy eating in this at-risk group.

A disadvantage of using a telephone method includes lack of visual cues, e.g. changes in body language, inability to build rapport [63–65]. However, using a telephone to conduct interviews was necessary as participants were recruited across a wide geographical area (metropolitan and non-metropolitan regions of Victoria, Australia). Telephone interviewing is a beneficial mode of collecting qualitative data as the views of participants in more remote regions can be included [58, 66, 67]. Also, one-on-one interviews may provide greater depth than possible in other forms of qualitative research such as focus groups, as concentrated time is spent with each participant [65], and participants may be more relaxed and willing to talk freely [68].

Given only one coder examined data, the potential for bias in identification and interpretation of themes was attenuated by the co-authors providing feedback during the data analysis stage. Findings revealed the importance of parents and the home food environment as key influences on diet among socioeconomically disadvantaged adolescents. As the present investigation is limited to adolescent perceptions, future research should include parental perceptions as well.

There are several strengths to the present study. The qualitative design provided detailed insights into a number of intrapersonal, social and environmental strategies supportive of disadvantaged adolescents’ eating behaviours as described by adolescents experiencing socioeconomic disadvantage. The perspectives of disadvantaged adolescents residing in metropolitan and non-metropolitan regions were represented. The use of a qualitative data collection method also provided rich descriptive details of perceived relevant and practicable initiatives that could be implemented in interventions aiming to improve eating behaviours among disadvantaged adolescents.

## Conclusions

Future design of interventions aimed at improving eating behaviours among disadvantaged adolescents need to take a multi-faceted approach, promoting intrapersonal strategies including increasing adolescent awareness about healthy eating and greater cooking involvement; social strategies could include increasing peer and parental support for and role modelling of healthy eating, and particularly, increasing availability of and access to nutritious foods while decreasing that of unhealthy foods in homes and schools. A unique contribution of this study is that particular emphasis should be placed on strategies related to increasing parental involvement and improving the food environment in adolescents’ homes to improve eating behaviours of socioeconomically disadvantaged adolescents. Research in the future should explore parental perceptions of such strategies.

## Abbreviations

SEP: Socioeconomic position
SEIFA: Socioeconomic Index for Areas
SMS: Short Message Service
